# A new genomic library of melon introgression lines in a cantaloupe genetic background for dissecting desirable agronomical traits

**DOI:** 10.1186/s12870-016-0842-0

**Published:** 2016-07-08

**Authors:** Gorka Perpiñá, Cristina Esteras, Yves Gibon, Antonio J. Monforte, Belén Picó

**Affiliations:** Instituto de Conservación y Mejora de la Agrodiversidad, Universitat Politècnica de València (COMAV-UPV), Camino de Vera s/n, 46022 Valencia, Spain; UMR1332 Biologie du Fruit et Pathologie and Plateforme Métabolome, INRA-Bordeaux and Bordeaux University, 71 av. Edouard Bourlaux, 33140 Villenave d’Ornon, France; Instituto de Biología Molecular y Celular de Plantas (IBMCP) UPV-CSIC, Ciudad Politécnica de la Innovación Edificio 8E, Ingeniero Fausto Elio s/n, 46022 Valencia, Spain

**Keywords:** Melon, Introgression line, Charentais, Fruit quality, Soluble solids concentration, QTLs

## Abstract

**Background:**

Genomic libraries of introgression lines (ILs) consist of collections of homozygous lines with a single chromosomal introgression from a donor genotype in a common, usually elite, genetic background, representing the whole donor genome in the full collection. Currently, the only available melon IL collection was generated using Piel de sapo (var. *inodorus*) as the recurrent background. ILs are not available in genetic backgrounds representing other important market class cultivars, such as the *cantalupensis*. The recent availability of genomic tools in melon, such as SNP collections and genetic maps, facilitates the development of such mapping populations.

**Results:**

We have developed a new genomic library of introgression lines from the Japanese cv. Ginsen Makuwa (var. *makuwa*) into the French Charentais-type cv. Vedrantais (var. *cantalupensis*) genetic background. In order to speed up the breeding program, we applied medium-throughput SNP genotyping with Sequenom MassARRAY technology in early backcross generations and High Resolution Melting in the final steps. The phenotyping of the backcross generations and of the final set of 27 ILs (averaging 1.3 introgressions/plant and covering nearly 100 % of the donor genome), in three environments, allowed the detection of stable QTLs for flowering and fruit quality traits, including some that affect fruit size in chromosomes 6 and 11, others that change fruit shape in chromosomes 7 and 11, others that change flesh color in chromosomes 2, 8 and 9, and still others that increase sucrose content and delay climacteric behavior in chromosomes 5 and 10.

**Conclusions:**

A new melon IL collection in the Charentais genetic background has been developed. Genomic regions that consistently affect flowering and fruit quality traits have been identified, which demonstrates the suitability of this collection for dissecting complex traits in melon. Additionally, pre-breeding lines with new, commercially interesting phenotypes have been observed, including delayed climacteric ripening associated to higher sucrose levels, which is of great interest for Charentais cultivar breeding.

**Electronic supplementary material:**

The online version of this article (doi:10.1186/s12870-016-0842-0) contains supplementary material, which is available to authorized users.

## Background

Melon (*Cucumis melo* L., 2n = 2 × = 24) is one of the most economically important fruit crops worldwide, with a current total world production of over 29 million tons [1]. For that reason, the development of new cultivars, not only with higher yields, but also with higher fruit quality standards and with attractive traits for consumers, is essential. Over the last few decades, biotechnological strategies have become indispensable tools for modern and efficient breeding in this crop.

Several transcriptome sequencing projects have been carried out in melon using Next Generation Sequencing (NGS) technologies in a set of genotypes representing the diversity of the species [2, 3]. The data generated have enabled the identification of large SNP (Single Nucleotide Polymorphism) and SSR (Single Sequence Repeats) collections (available at the [4]), which have facilitated the construction of consensus saturated maps [5, 6]. Some of these collections have been implemented in high-throughput genotyping platforms and have been used for genetic diversity and association studies [7, 8]. Some of these markers have also been used to anchor genetic and physical maps [6] on the melon genome sequence [9–11]. Other genomic tools available for melon research and breeding include microarrays [12, 13] as well as TILLING and EcoTILLING platforms [14–17]. These tools have allowed the genetic dissection of both simple and complex traits [18–26].

In this context, introgression line (IL) generation is an excellent breeding strategy for incorporating exotic natural variation into modern breeding programs. ILs are generated by backcrossing, starting from an F1 cross between one selected donor genotype, usually exotic or wild germplasm, and a common genetic background, usually an elite cultivar. Marker-assisted selection (MAS) of lines with target-donor introgressions and recurrent genetic background is performed in each generation [27]. The existing genomic tools and the use of genotyping platforms highly increases the efficiency of MAS, significantly reducing the number of backcross generations necessary to generate a collection of ILs that have single introgressions and which represent the entire donor genome [28]. Apart from the introduction of new variability into crops for breeding purposes, ILs also facilitate the detection of new QTLs. ILs have been developed in many crops, such as tomato [28–31], barley [32], lettuce [33] and rice [34], among others.

Melon is the most polymorphic cucurbit species [35, 36], showing impressive diversity in important commercial traits, such as fruit morphology, ripening behavior and organoleptic and nutritional fruit quality. *C. melo* is subdivided into two subspecies: ssp. *melo* and ssp. *agrestis,* which can be further divided into 16 botanical groups [37]: *inodorus*, *cantalupensis, reticulatus*, *ameri, chandalack, adana, flexuosus*, *chate*, *dudaim* (within ssp. *melo*); and *acidulus*, *conomon*, *makuwa*, *chinensis*, *momordica*, *chito* and *tibish* (the latter two of which have been reclassified according to molecular studies to be within the ssp. *agrestis* [7, 8]).

The most important commercial cultivars belong to the *inodorus, reticulatus* and *cantalupensis* groups, while cultivars belonging to the subsp. *agrestis* are considered “exotic” for applied breeding. The only IL collection reported in melon to date was derived from the cross of the Spanish cultivar Piel de Sapo (subsp. *melo* var. *inodorus*) and the Korean donor accession PI 161375 (Songwan Charmi; subsp. *agrestis* var. *chinensis*), which carries several pest- and disease-resistant genes [38]. This first IL population has been used for different breeding purposes: root structure-related traits [21], fruit quality, including fruit weight, shape and flesh color [20, 39], sugar and organic acid content [40], aroma profile [41], climacteric behavior [24, 42] and resistance to pathogens, such as Cucumber Mosaic Virus (CMV) [43, 44].

However, until now there have been no melon ILs generated in a *cantalupensis* genetic background. In the current article, we report the development of a new IL population derived from the cross between the French cultivar Vedrantais (VED), a Charentais type, as recurrent parent (subsp. *melo* var. *cantalupensis*) and the Japanese Ginsen makuwa (MAK) cultivar (subsp. *agrestis* var. *makuwa*). This donor genotype was selected from the melon core collection built in the framework of a previous project (MELRIP 2007–2010) [7, 15] due to its interesting quality traits, especially its higher sugar content compared to most *agrestis* types [8]. The IL population has been used to identify QTLs related to fruit morphology, ripening behavior and organoleptic and nutritive quality. It also provides pre-breeding lines with new phenotypes in a Charentais genetic background that could be useful for the development of new cultivars.

## Results and discussion

### Development of the IL population

The two parents used to generate the IL population were the cultivar Vedrantais (VED) (*C. melo* subsp. *melo* var. *cantalupensis*, Charentais type) as recurrent parent and Ginsen makuwa (MAK) (*C. melo* subsp. *agrestis* var. *makuwa*) as donor parent (Additional file 1). Fifteen BC1 plants derived from the cross VED x MAK were backcrossed to the recurrent parent, thus producing fifteen BC2 families, each one with twenty eight plants. The 420 BC2 seedlings were genotyped with the Sequenom array with 154 SNPs [3, 8, 9], from nine to twenty per chromosome (Additional file 2). Seventy-five BC2 seedlings with the highest proportion of the recurrent (VED) genome and with MAK introgressions covering the entire donor genome were selected. These BC2 were transplanted to the greenhouse for phenotyping and twenty-two of them were backcrossed to VED in order to generate the BC3 population. This BC2 set averaged 6.5 introgressions/plant and 88.2 % of the VED background genome (ranging from 76.2-96.3 %), representing twice the entire MAK genome.

A total of 363 seedlings of the BC3 population were genotyped with the same Sequenom array. One hundred BC3 seedlings were selected according to their genotype (following the same criteria as in the BC2) and were transplanted to the greenhouse for phenotyping. Thirty-three were selected and used to generate the IL population. These selected BC3 plants presented an average of 3.4 introgressions/plant and 93.2 % of the VED background genome (range 84.1–98.2 %). The early selection in a large number of plants greatly facilitated the recovery of the recurrent genetic background (an average increase of 5 %), and the reduction of the number of introgressions per plant (to about 3) in one backcross generation.

Twenty-four of the selected BC3 plants had three or fewer MAK introgressions/plant. These were selfed to produce the BC3S1 generation. After BC3S1 seedling screening with appropriate SNP markers, it was possible to obtain single homozygous MAK introgression genotypes in most cases. Ninety-six BC3S1 plants were selected according to their genotype and transplanted to the greenhouse for phenotyping. A second round of selfing (generation BC3S2) was necessary in some cases to fix certain heterozygous markers and generate enough seeds for further assays.

The remaining nine selected BC3 plants had four or more introgressions, so they were used to produce an additional backcross generation (BC4). Thirty four BC4 plants with single or double introgressions were then selected and selfed once or twice to produce BC4S1 and BC4S2 plants with single or double MAK homozygous introgressions. The selection of plants with homozygous target introgressions in all these generations (BC3S1, BC3S2, BC4, BC4S1 and BC4S2) was carried out with the SNPs of the corresponding introgressions by High Resolution Melting.

A first core collection of 27 ILs, mostly with a single introgression and a few with double introgressions, all homozygous, representing most of the MAK genome (Additional file 3), were subjected to further phenotyping in three trials, along with the VED and MAK parents and their F1. This set represents the MAK genome quite well, and has an appropriate size for performing accurate phenotyping with climacteric fruits. This IL collection has an average of 1.3 introgression/IL, representing 95.4 % of the VED background genome (range 89.8–99.1 %). A total of 37 bins were defined with an average of 2.8 bins/chromosome. The average size of the introgressions was 30 cM. Some regions of the MAK genome in chromosomes 1, 4, 5, 7 and 8 were not represented (10 %) (Additional file 3).

### Parent phenotypes

The two parents showed clear differences in a number of traits related to flowering time, fruit morphology, fruit ripening behavior and traits related to organoleptic and nutritive value, such as flesh color and sugars content. Additional file 4 depicts the mean values and standard deviations, along with ANOVA results for means comparison of both parents, VED and MAK, and their F1 for each studied trait in the three trials in which they were phenotyped along with the ILs. MAK showed earlier and more female flowering than VED plants (with an average across environments of 3.6 versus 1.6 female flowers per plant 30 days after the opening of the 1^st^ flower, NFeF30), but no differences were observed for the male flowering pattern (8.5 versus 7.5 male flowers per plant, NMaF30). VED fruits were significantly heavier (average Fruit weight (FW) 755.9 g) than MAK fruits (243.9 g), which yielded more elongated fruits with higher percentages of seminal cavity (Fruit shape (FS) 1.1 versus 0.92, and seminal cavity/fruit diameter ratio (CW) 0.61 versus 0.45, respectively for MAK and VED). The formation of an abscission layer at the time of ripening (AL) and the occurrence of external aroma in mature fruits (AR) were present in VED and absent in MAK, these being indicators of the climacteric behavior of the Charentais type. Differences in other traits, such as rind thickness (RTh, 4.2 mm versus 1.7 mm in VED and MAK, respectively) and rind netting (Net, present in VED and absent in MAK) were also observed (Additional file 4). Regarding the traits related to flesh quality, VED is orange-fleshed, whereas MAK is white-fleshed (Additional file 1), with different values for the colorimeter parameters, such as higher luminosity Hl, negative a* values and lower b* values in the MAK parental (FCHl 64.7, FCa −2.2, FCb 10.6 versus FCHl 53.6, FCa 11.1 and FCb 23.8, in MAK and VED, respectively) (Additional file 4). Both parents are sweet melons, with similarly high soluble solids content (SSC) in the fruits (11.2° versus 11.7° brix degrees for VED and MAK, respectively) and similar amounts of sucrose and fructose, but with significantly lower levels of glucose in the MAK fruits (Suc 248.2, Gluc 85.9 and Fruc 87.5 versus Suc 232.7, Gluc 46.0 and Fruc 50.9 in μmol/gFW eq. Hexose) (Additional file 4).

### Association analysis in backcross families

Table 1 shows significant associations (at *p* < 0.005) identified by TASSEL, by both GLM and MLM analysis, and those identified only by GLM, but which were also identified in the IL analysis described below.

**Table 1:** Association analysis performed with TASSEL v.5, using GLM and MLM models in the BC2, BC3 and BC3S1 populations

					GLM			MLM				
Trait	Population	Marker	Chromosome	Position (cM)	*p*	Marker_Rsq	Allelic effect	*p*	Marker_Rsq	Allelic effect	Parental that increase the trait value	IL
FS	BC3	PSI_41-B07	11	27.6	0.003409612	0.08	−0.07				MAK	MAK 11.2
	BC2	CMPSNP30	11	66.0	0.004866798	0.09	−0.06				MAK	MAK 11.2
	BC3	CMPSNP30			3.45E-04	0.12	−0.09	0.004918	0.08	−0.11	MAK	MAK 11.2
FF	BC2	CMPSNP249	7	11.3	9.65975E-05	0.19	−2.47				MAK	MAK 7.2
	BC3	CMPSNP249			0.001718059	0.10	−1.87				MAK	MAK 7.2
	BC2	CMPSNP262	7	30.5	0.001993046	0.12	−2.47				MAK	MAK 7.2
	BC3	CMPSNP262			1.27E-05	0.18	−2.54				MAK	MAK 7.2
	BC3	CMPSNP1009	7	32.1	1.27E-05	0.18	−2.54				MAK	MAK 7.2
	BC3	CMPSNP287	7	35.3	2.77E-04	0.13	−2.15				MAK	MAK 7.2
	BC3	CMPSNP56	7	43.3	0.00119487	0.10	−1.69				MAK	MAK 7.2
	BC2	CMPSNP65	10	14.4	8.56312E-08	0.33	−3.09	0.004796	0.11	−2.22	MAK	MAK 10.1
	BC3	CMPSNP65			0.004653477	0.08	−1.41	0.004401	0.07	−1.28	MAK	MAK 10.1
FCHl	BC3S1	CMPSNP1077	9	19.2	0.002510183	0.09	−19.50	0.003482	0.10	−19.00	MAK	MAK 9.2
FCa	BC3	AI_14-H05	2	40.6	0.001120465	0.10	−2.73	0.000950	0.12	−3.59	MAK	MAK 2.1
	BC3S1	CMPSNP320	9	20.8	1.86E-10	0.38	15.02				VED	MAK 9.2
	BC3	CMPSNP144	9	22.4	9.75E-04	0.11	2.25				VED	MAK 9.2
	BC3S1	CMPSNP144			2.10E-10	0.39	17.86				VED	MAK 9.2
	BC3	CMPSNP1035	9	33.6	9.53E-05	0.15	2.31				VED	MAK 9.2
	BC3S1	CMPSNP1035			2.68E-16	0.51	17.98				VED	MAK 9.2
	BC3	CMPSNP159	9	36.8	2.20E-04	0.13	2.20				VED	MAK 9.2
	BC3S1	CMPSNP159			1.35E-13	0.48	18.11				VED	MAK 9.2
	BC3S1	CMPSNP1133	9	59.2	3.51E-12	0.44	17.64	0.003116	0.13	6.11	VED	MAK 9.2
	BC3S1	CMPSNP890	9	64.0	9.90E-10	0.36	17.64				VED	MAK 9.2
FCb	BC3S1	CMPSNP320	9	20.8	2.58E-06	0.24	6.09				VED	MAK 9.2
	BC3S1	CMPSNP144	9	22.4	5.49E-06	0.23	8.06				VED	MAK 9.2
	BC3S1	CMPSNP1035	9	33.6	3.57E-12	0.40	8.11	0.000031	0.20	9.20	VED	MAK 9.2
	BC3S1	CMPSNP159	9	36.8	2.21E-06	0.25	6.78				VED	MAK 9.2
	BC3S1	CMPSNP1133	9	59.2	1.77E-06	0.25	7.86				VED	MAK 9.2
	BC3S1	CMPSNP890	9	64.0	4.56E-06	0.23	7.86				VED	MAK 9.2
SSC	BC2	CMPSNP731	1	80.4	0.000782493	0.14	1.62				VED	
	BC3	CMPSNP731	1	80.4	0.000468736	0.12	2.07				VED	
	BC3S1	CMPSNP731	1	80.4	0.002121734	0.10	4.11				VED	
	BC3	CMPSNP204	1	86.8	0.001202906	0.10	2.32				VED	
	BC2	CMPSNP65	10	14.4	1.65953E-06	0.27	−3.26	0.004937	0.11	−2.22	MAK	MAK 10.1
SUC	BC3S1	CMPSNP731	1	80.4	0.004606327	0.11	117.72				VED	MAK 1.2
	BC3S1	CMPSNP204	1	86.8	0.00454631	0.11	117.19				VED	MAK 1.2
GLUC	BC3S1	60 k41.243	5	73.4	0.004036775	0.12	−81.06	0.004423	0.13	−84.40	MAK	MAK 5.2
	BC3S1	AI_13-H12	5	89.4	0.004036775	0.12	−81.06	0.004423	0.13	−84.40	MAK	MAK 5.2
FRUC	BC3S1	CMPSNP1133	9	59.2	0.000596137	0.20	37.26	0.004122	0.17	20.61	VED	
	BC3S1	CMPSNP890	9	64.0	0.000596137	0.20	37.26	0.004122	0.17	20.61	VED	

Only associations at *p* < 0.005 observed in both GLM and MLM analysis or in GLM and IL analysis (Additional file 5, Figs. 2, 3, 4, 5 and 6) are shown. For each trait (*FS* fruit shape index, *FF* flesh firmness, *FCHl, FCa and FCb* L, a* and b* Hunter coordinates of flesh color, *SSC* soluble solid concentration in flesh, *SUC, GLUC and FRUC* sucrose, glucose and fructose content in flesh) with significant association, the backcross population where it was identified, the significant marker with its chromosome and genetic position in cM, the statistical significance of the association (p), the percentage of phenotypic variance explained by the marker (R^2^), the allelic effect (negative when MAK alleles increase the trait value), and the IL that showed a significant effect in the trait and carried the marker introgressed from MAK (identified with the Dunnet’s test in the IL analysis), are also indicated

GLM identified several markers in chromosome 11 associated to FS. The SNP CMPSNP30 (66 cM) was significant in two populations, BC2 and BC3 (*R*^*2*^ = 9.3 and 12.3 %); it was also significant in the BC3 according to MLM (*R*^*2*^ = 8.4). In all cases, MAK alleles increased FS values, resulting in more elongated fruits. CMPSNP65 on chromosome 10 (14.4 cM) was found to be associated with FF in both the BC2 and BC3 populations using both GLM (*R*^*2*^ = 32.7–7.9 %) and MLM (*R*^*2*^ = 11.4 and 7.3 %) analysis, with the MAK alleles resulting in firmer flesh (Table 1). This characteristic is associated with the ripening behavior and may be related to postharvest conservation. The same effect of MAK alleles was identified by GLM in both populations on chromosome 7 (SNPs CMPSNP249 and CMPSNP262 at 11.3 cM and 30.5 cM). However, this association could not be confirmed by MLM.

Flesh color was associated with several genomic regions (Table 1). The most important effects were observed on chromosome 9, significant in populations BC3 and BC3S1, with a main effect in the interval between markers CMPSNP1035-CMPSNP1133 (33.6–59.2 cM), with the GLM/MLM models explaining up to 51.1/13.4 % and 40.4/20.1 % of the variation found for the a* and b* parameters (FCa and FCb). Also found in chromosome 9 was a significant association of CMPSNP1077 with Hl, in both GLM and MLM models. VED alleles increased the a* and b* parameters and reduced Hl, which resulted in orange-fleshed fruits. Additionally, SNP AI_14-H05 (located on chromosome 2 at 40.6 cM) was found to be associated to FCa by both GLM and MLM analysis in the BC3 population (*R*^*2*^ = 10.4 and 11.9 %, respectively), but interestingly, this time the MAK alleles increased FCa.

Four regions that affected variation of the sugars content were detected on chromosomes 1, 5, 9 and 10 (Table 1). The region on chromosome 1 (CMPSNP731, 80.4 cM) was only detected with GLM, with the MAK alleles decreasing flesh soluble solids content (SSC) in all backcross generations (BC2, BC3 and BC3S1) and sucrose content (SUC) in BC3S1. Even though this association could not be verified by MLM, it was validated later on in the IL analysis (see below). On chromosome 9, MAK alleles of CMPSNP1133 and CMPSNP890 (positions 59.2 and 64.0 cM, respectively) were associated with a reduction in fructose content by both GLM and MLM analysis. More interestingly, MAK alleles of CMPSNP65 (on chromosome 10, position 14.4 cM) and of 60 k41243 and AI_13-H12 (on chromosome 5, positions 73.4 and 89.4 cM) were associated to an increase of SSC and glucose content in BC2 and BC3S1, respectively, using both GLM (*R*^*2*^ = 27.1 and 11.4 %) and MLM (*R*^*2*^ = 11.5 and 12.7 %) analysis.

### QTL analysis in the IL population

**Fig. 1:**
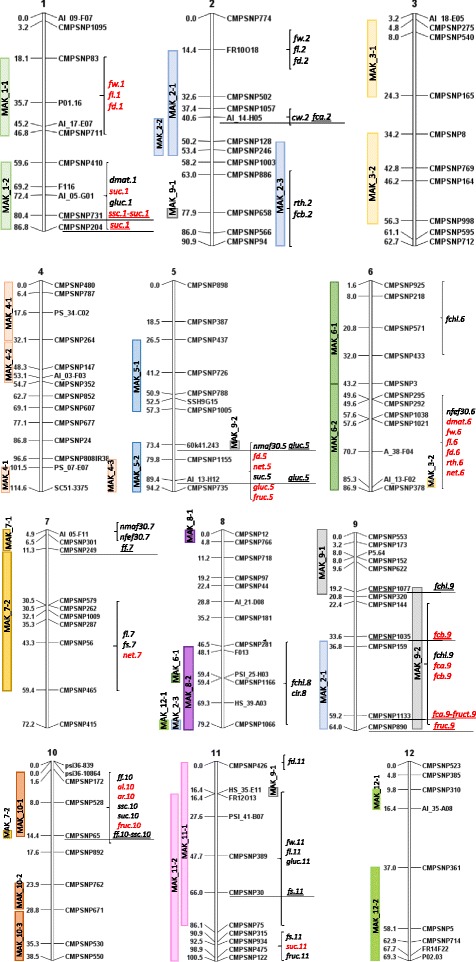
QTL locations in the map of [9]. Markers found to be associated in the backcross populations (underlined) are indicated (for each associated region all the markers significant with both GLM and MLM analysis and those markers significant with MLM and having the highest R2 values are shown). QTLs found in the ILs assay with the Dunnett’s test in at least two trials are shown in brackets. In red QTLs in which MAK alleles decrease the value of the trait and in black those in which MAK alleles increase the value of the trait. Color bar at the left of the chromosomes show the MAK introgressions of each IL

**Fig. 2:**
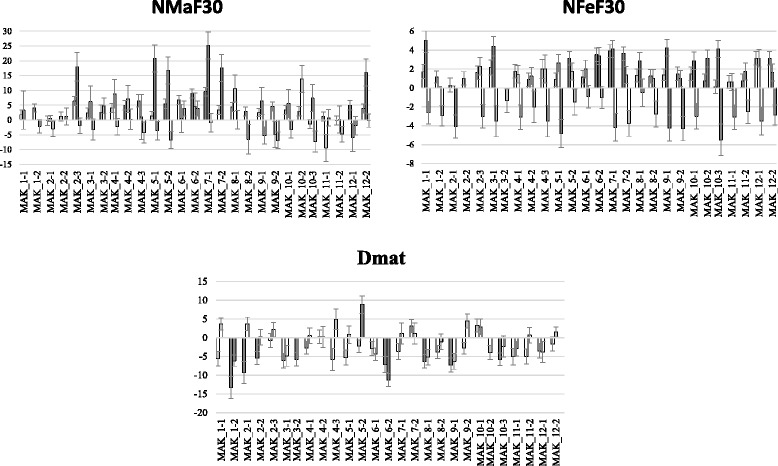
Comparison of the means of the set of ILs with the mean of the recurrent parent (VED) using the Dunnett’s test. The means and standard errors are shown for each trial (Paiporta 2015, UPV 2015 and Paiporta 2014). Gray bars show significantly different (*p* < 0.05) IL and VED means. Traits evaluated are: NMaF30 and NFeF30 = Number of male/female flowers 30 days after the appearance of the first flower and Dmat = days to maturity. For Dmat, only two trials are shown (UPV 2015 and Paiporta 2014)

**Fig. 3:**
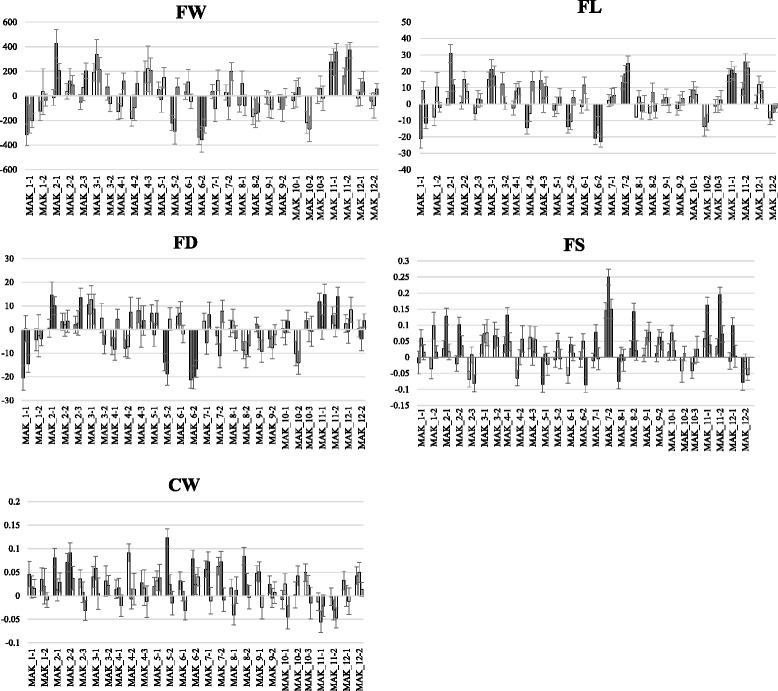
Comparison of the means of the set of ILs with the mean of the recurrent parent (VED) using the Dunnett’s test. The means and standard errors are shown for each trial (Paiporta 2015, UPV 2015 and Paiporta 2014). Gray bars show significantly different (*p* < 0.05) IL and VED means. Traits evaluated are: FW = fruit weight in grams, FL = fruit length in mm, FD = fruit diameter in mm, FS = fruit shape as the ratio between fruit length and fruit diameter and CW = cavity width (as the ratio between the width of the seminal cavity and the fruit diameter)

**Fig. 4:**
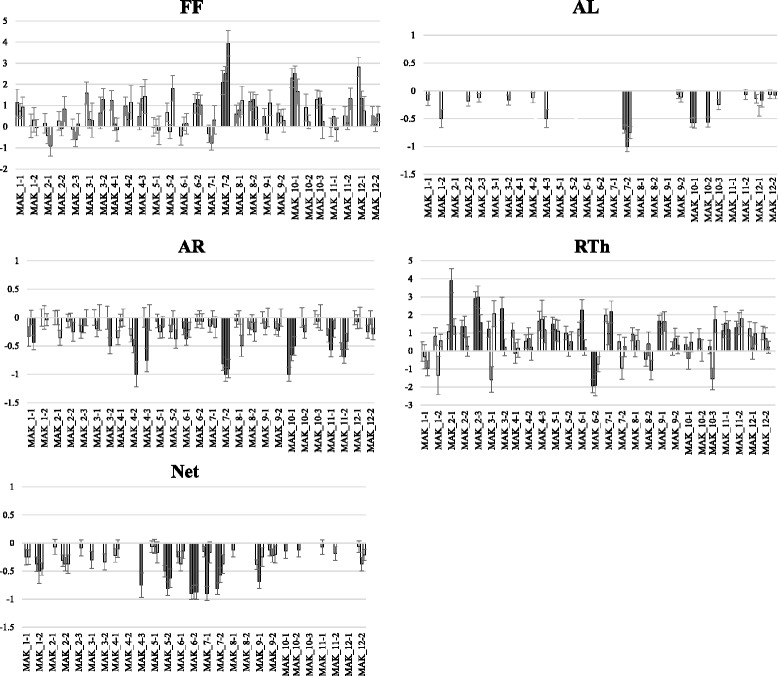
Comparison of the means of the set of ILs with the mean of the recurrent parent (VED) using the Dunnett’s test. The means and standard errors are shown for each trial (Paiporta 2015, UPV 2015 and Paiporta 2014). Gray bars show significantly different (*p* < 0.05) IL and VED means. Traits evaluated are: FF = flesh firmness in kg/cm^2^, AL = presence or absence of abscission layer, AR = presence or absence of external aroma, RTh = rind thickness in mm and Net = presence or absence of rind netting

**Fig. 5:**
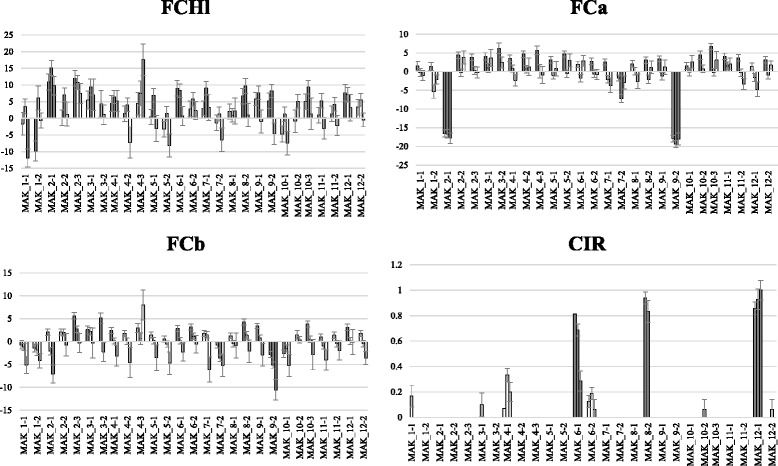
Comparison of the means of the set of ILs with the mean of the recurrent parent (VED) using the Dunnett’s test. The means and standard errors are shown for each trial (Paiporta 2015, UPV 2015 and Paiporta 2014). Gray bars show significantly different (*p* < 0.05) IL and VED means. Traits evaluated are: Hunter coordinates, FCHl = flesh color luminosity, FCa = flesh color a* parameter, FCb = flesh color b* parameter and CIR = color of the inner rind scored visually

**Fig. 6:**
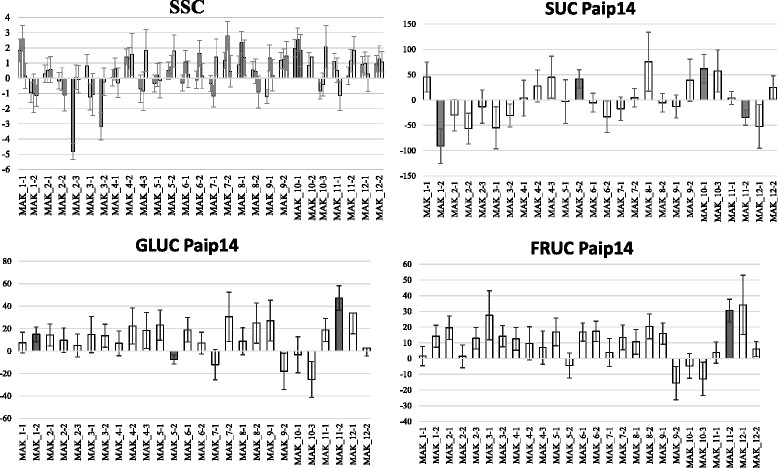
Comparison of the means of the set of ILs with the mean of the recurrent parent (VED) using the Dunnett’s test. The means and standard errors are shown for each trial (Paiporta 2015, UPV 2015 and Paiporta 2014). Gray bars show significantly different (*p* < 0.05) IL and VED means. Traits evaluated are: SSC = soluble solids content in Brix degree, SUC = sucrose, GLUC = glucose and FRUC = fructose, all in μmol/gFW eq. Hexose. For SUC, GLUC and FRUC, the mean of Paiporta 2014 is presented

The Dunnett’s test of the IL population phenotyped in the three environments allowed the detection of a number of QTLs (Additional file 5 and Figs. 1, 2, 3, 4, 5 and 6) as described in the next section.

#### Flowering and maturity time

The IL population showed high variability for flowering traits (NMaF30 ranging from 1.1 to 36.1 and NFeF30 from 0 to 5.5) (Additional file 4). We found low to moderate heritabilities for both traits (h^2^ = 0.26 to 0.40 and 0.15 to 0.26, for male and female flowering, respectively) and significant G x E interaction (19.6 and 18.3 %, respectively). This interaction was probably a consequence of the high temperatures reached in the Pap14 trial, which accelerated plant development, making more frequent pruning necessary, which in turn likely affected flowering scoring. Also, the time from pollination to maturity was highly variable among the ILs (DMat from 27 to 52.1 days). This trait was measured only in the two assays where hand pollination was used (UPV15 and Paip14). Heritabilities for this trait were slightly higher (h^2^ = 0.26 to 0.48), and G × E interaction represented 10.1 % of total variance.

Despite the high interaction effect, several ILs showed consistent significant differences with the VED recurrent parent in at least two localities (Fig. 2), thereby defining 6 QTLs (*nmaf30.5, nmaf30.7, nfef30.6, nfef30.7, dmat.1* and *dmat.6*) (Additional file 5, Fig. 1). MAK_5-2 produced more male flowers, whereas MAK_6-2 developed a higher number of female flowers. Interestingly, MAK_7-1 produced more of both male and female flowers (Fig. 2). Apart from the effect on the number of female flowers, MAK_6-2 showed a ripening cycle that was shorter than that of VED. These two traits found together in MAK_6-2 are interesting, as they could be useful for developing cultivars with abundant and early yield. The effect of a shorter cycle was also observed in MAK_1-2 (Fig. 2).

#### Fruit morphology

Traits related to fruit size and shape (fruit weight, length, diameter and shape (FW, FL, FD and FS)), presented moderate heritability (h^2^ = 0.38 to 0.55), and all had a low or non-significant G x E interaction (6.2–8.1 %) (Additional file 4). FS was the trait with the lowest environmental effects, which was consistent with the information reported in previous studies [20]. Another trait related to fruit morphology is percentage of fruit cavity (CW). CW had lower heritability (h^2^ = 0.08 to 0.29) and higher G x E effects (11.8 %) (Additional file 4).

**Fig. 7:**
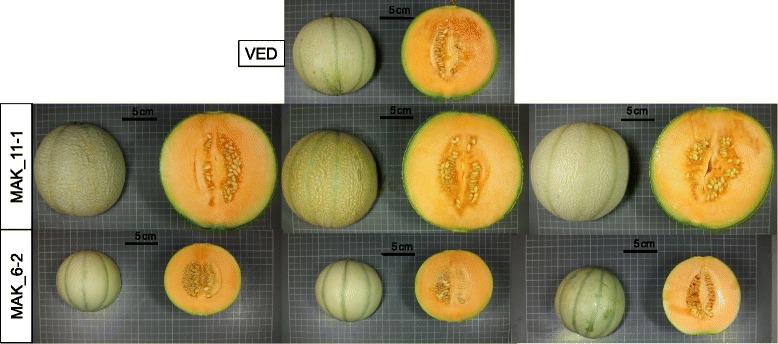
Effect of MAK introgressions in chromosomes 11 and 6 affecting fruit size. *Top*: VED parental (FW = 614–933 g; FL = 98.6–112.7 mm; FD = 107.6–126.3 mm). Middle: melons of MAK_11-1 (FW = 971.2–1208.1 g; FL = 117.30–133.3 mm; FD = 122.5–127.4 mm). *Bottom*: melons of MAK_6-2 (FW = 366.9–579.2 g; FL = 75.8–99.7 mm; FD = 89.8–106.1 mm). Both lines show significant differences in FW, FL and FD with VED in two or three trials

FW was positively correlated to fruit length and diameter (FL and FD) in all localities (*r* = 0.77 to 0.93) (Additional file 6). In fact, most of the ILs that showed significant effects on FW also showed effects in both FL and FD (Fig. 3). MAK_1-1 and MAK_6-2 significantly decreased FW in at least two environments (from 32.5 to 46.7 %), whereas MAK_2-1 and MAK_11-1 increased FW (from 33.3 to 58.1 %), even though MAK showed smaller fruits than VED, which demonstrates the power of the current population to uncover hidden genetic variability. These four ILs also presented significant effects on FL and FD, with MAK alleles having the same direction of effect. Therefore, these changes in FW were due to variation in two dimensions at the same time and had no effect on fruit shape. These lines defined four FW QTLs that co-localize with those of FL and FD (*fw.1-fl.1-fd.1, fw.2-fl.2-fd.2, fw.6-fl.6-fd.6.* and *fw.11-fl.11-fd.11*) (Fig. 1 and Additional file 5). Representative fruits of the two lines with the largest effects on FW, MAK_6-2 and MAK 11–1, are shown in Fig. 7.

**Fig. 8:**
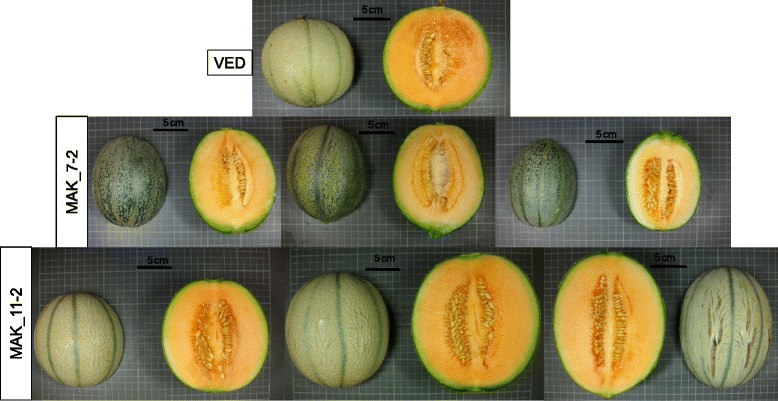
Effect of MAK introgressions in chromosomes 7 and 11 affecting fruit shape. *Top*: VED parental (FS = 0.89–0.94). *Bottom*: melons of the line MAK_7-2 (FS = 1.08–1.14) and melons of the line MAK_11-2 (FS: 0.97–1.08)

FW was not correlated to Fruit shape (FS) (*r* = 0.18 or non-significant). However, FS was positively correlated to FL (*r* = 0.53 to 0.59) (Additional file 6), as previously observed [20, 45]. MAK_7-2 and MAK_11-2 yielded fruits with an increase in the FS ratio, which were more elongated than VED (between 12.5 and 25 % longer), but had no significant variation in diameter (Figs. 3 and 8), which defined QTLs for FL and FS in these regions (*fl.7-fs.*7 and *fl.11-fs.11*) (Fig. 1 and Additional file 5).

**Fig. 9:**
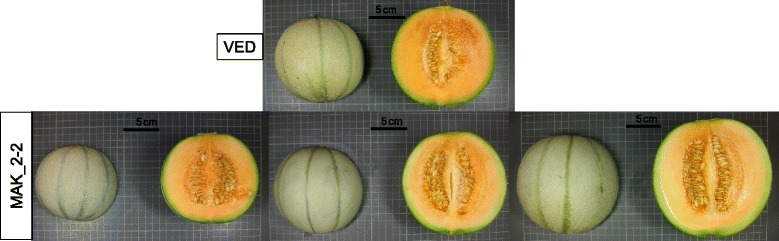
Wider cavities found in fruits of MAK_2-2 (*Bottom*: CW = 0.50.–0.54) compared to the recurrent VED parental (*Top*: CW = 0.42–0.49)

One QTL involved in variation of CW was identified in chromosome 2, *cw.2*. Fruits of MAK_2-2 had higher CW values (from 16.7 to 20.2 %) than the VED parental line (Fig. 3). The effect of this QTL is shown in Fig. 9. The introgression of MAK_2-2 totally overlapped with that of MAK_2-1 and partially with that of MAK_2-3 (Additional file 3). MAK 2–1 also had CW values that were higher than those of VED in one environment, whereas MAK_2-3 did not differ from the recurrent parent in this trait (Fig. 3), thus suggesting that this QTL is in the region that is common to both MAK_2-1 and MAK_2-2.

In summary, with the current IL population we have identified several QTLs involved in FW, FS and CW. Nearly 60 and 30 QTLs have been described to date for FW and FS [6, 46], respectively. In the current report, QTLs *fw.2* and *fw.11* may correspond to the metaQTLs *FWQM2* and *FWQM11* described by [46]. This study [46] suggested that members of the CNR/FW2.2 and SlKLUH/FW3.2 gene families are strong gene candidates for melon FW QTLs. Among the QTLs detected with the current IL collection, the *fw.6* and *fw.11* chromosomal regions include the CNR melon member *CmCNR-6* and the SlKLUH melon member *CmCYP78A-4*, respectively. However, further studies using subILs with smaller introgressions are necessary to analyze the contribution of these genes.

FS QTLs have previously been mapped in all chromosomes, except in chromosome 5, and metaQTLs had been defined on chromosomes 1, 2, 8, 11 and 12. In the current report, *fs.11* may correspond to the metaQTL *FSQM11*. Regarding the QTL defined on chromosome 7, several FS QTLs had been detected previously in that genomic region, mainly in PI161375 (a Korean accession closely related to *makuwa* cultivars, [7]) × “Piel de Sapo” (*inodorus* type) populations, suggesting that this QTL may be specific to Far-Eastern melon cultivars. The candidate genes *CmOFP-8* (member of the Ovate Family Proteins) and *CmSUN-16* (member of the *SUN* family) are located in the region of the QTL.

QTLs involved in fruit flesh content or cavity have been studied in a very small number of works. Nevertheless, [47] found QTLs for these traits on LG II, which could be allelic to the QTL observed in MAK_2-2.

#### Ripening behavior

Ripening behavior is one of the most important factors involved in fruit quality in melon. We analyzed three ripening-related traits: the presence of abscission layer, flesh firmness and external aroma (AL, FF and AR). All three traits presented moderate heritability (h^2^ = 0.30 to 0.5) with a strong genotype effect (which accounted for 29.2 to 35.9 % of the total variation), a low or non-significant environmental effect and moderate G × E interaction (9.1–11.5 %) (Additional file 4).

**Fig. 10:**
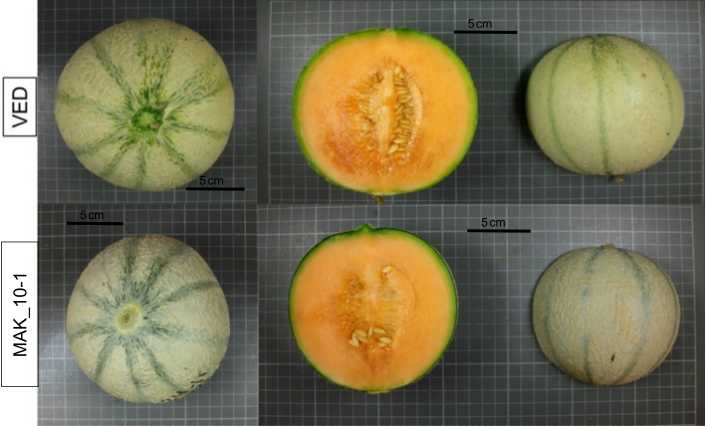
Fully mature fruits of VED with abscission layer (*top*) and of line MAK 10.1 (*bottom*) without abscission layer

Most ILs developed fully climacteric fruits, like VED, forming an abscission layer at the time of maturity; two exceptions were MAK_7-2 and MAK_10-1, in which most of the fruits did not form an abscission layer at full maturity (Figs. 4 and 10). MAK_10-1 has a single introgression, defining the QTL *al.10* (Fig. 1 and Additional file 5), whereas MAK_7-2 has a major introgression on chromosome 7, and also an additional one in the region of *al.10* (Additional file 3), which could be causing the effects on the abscission layer. Therefore, the possible presence of an *al* QTL on chromosome 7 needs to be confirmed by separating these two introgressions into independent lines.

Along with the lack of fruit abscission, MAK_7-2 and MAK_10-1 showed firmer flesh (FF increased about twofold compared to VED, 4.2 and 4.9 versus 2.0 kg/cm^2^) and less external aroma at maturity (*ff.10* and *ar.10*, Fig. 4), probably as a result of the different ripening behaviors, as these two traits have been reported to be influenced by climacteric/non-climacteric ripening [42, 48]. The same possible confounding effects between chromosome 7 and chromosome 10 introgressions on MAK_7-2 may be causing the increase in FF as well as the aroma decrease observed in this IL.

None of the QTLs found in this study correspond to previous QTLs associated to ripening. For example, the major genes *Al-3* and *Al-4* that control fruit abscission and autocatalytic ethylene production map in different chromosomes (3 and 9, respectively [49]). Additional QTLs involved in ethylene production and or in fruit flesh firmness [8, 24, 49, 50] map in different regions of chromosomes 1, 2, 3, 6, 11 and 12. [50] reported an increased FF in ILs with introgressions of chromosome 10, derived from the cross of PI161375 (whose climacteric behavior is similar to that of MAK) × “Piel de Sapo” (*inodorus,* non-climacteric), which is most likely allelic to the QTL detected in MAK_10-1 (*ff.1*0). The fruit flesh of the Piel de Sapo cultivar actually has a high FF value, so much so that increasing FF in this genetic background would probably not be necessary or even desirable, whereas an increase of FF in *cantalupensis* cultivars could be very interesting. Thus, delayed ripening and increased flesh firmness could extend fruit shelf life, which is a major objective for cantaloupe breeding.

#### Rind characteristics

Rind characteristics, such as rind thickness and netting (Rth and Net) can be associated with shelf life and resistance to storage and shipping. Despite this potential, the study of their genetics has been scarce [23, 42]. These traits have moderate heritability (h^2^ = 0.33–0.49) with important genotype contributions for both traits (29–35.8 %), and moderate and low E and G × E effects, in Rth and Net, respectively (Additional file 4).

**Fig. 11:**
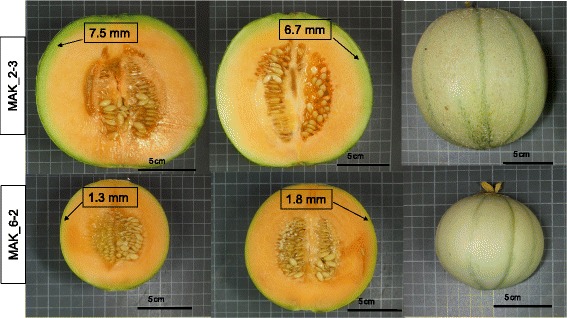
Variation in rind thickness observed in the IL Population. *Top*: Thicker rinds of line MAK_2-3 (Rth = 5.8–7.7 mm) and *Bottom*: thinner rinds of line MAK_6-2 (Rth = 1.3–3.5 mm). Right: detail of netted and non-netted rind

MAK_2-3 and MAK_6-2 produced fruits with thicker and thinner rinds, respectively, than VED (average of 6.6 mm and 2.6 mm versus 4.2 mm in VED, Figs. 4 and 11) in at least 2 environments. These lines defined the *rth.2* and *rth.6* QTLs (Fig. 1 and Additional file 5). *Rth.6* co-localized with *net.6* (Fig. 1, Additional file 5), which accounted for fruits of the IL MAK_6-2 being less netted than those of VED (Figs. 4 and 11). This phenotype can be seen as either a benefit, fruits with more edible flesh, or a liability, more problems in harvesting and storage. Two other QTLs were associated to netting reduction (*net*.5 and *net*.7) (Additional file 5), as observed in MAK_5-2 and MAK_7-2 fruits with reduced netting intensity (Fig. 4).

#### Flesh color

Flesh color is important as a consumer preference trait, and can also be associated to carotenoid content, which is related to the nutritional quality of the fruit. Our IL population showed important variability for flesh color, as was expected due to the contrasting phenotypes of the founder parents for this trait (Additional files 1 and 4). All the measured traits related to flesh color displayed a very strong genotype effect (29.1–75.7 %), with heritabilities from moderate in FCHl (h^2^ = 0.32 to 0.36) to high in the FCa and FCb (h^2^ = 0.60 to 0.83 and h^2^ = 0.43 to 0.47, respectively). The E effects (1.5–7.9 %) and G × E interactions (3.6–9.5 %) were low and even non-significant for the b* parameter (Additional file 4).

**Fig. 12:**
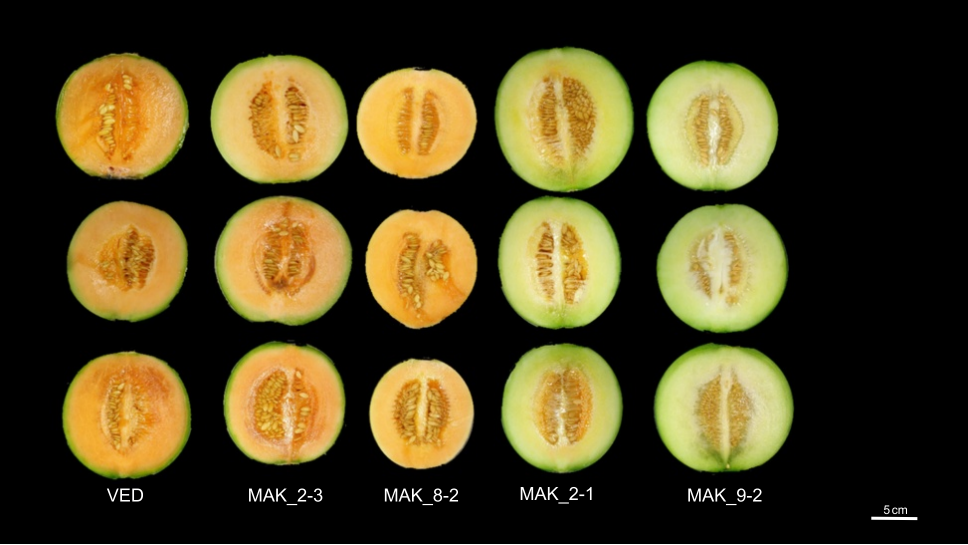
Variability in flesh color in different introgressions lines. Columns Left to right: VED, MAK_2-3, MAK_8-2, MAK_2-1 and MAK_9-2

The main effect in flesh color was observed in MAK_2-1 and MAK_9-2, which yielded fruits with green flesh (Fig. 12), with significantly lower and higher values for the a* and Hl parameters, respectively, in the three environments (Fig. 5). Fruits of MAK 9_2 also had b* values that were significantly lower than VED. These lines define a major QTL affecting flesh color in chromosome 9 (*fchl.9-fca.9-fcb.9*) (Additional file 5). The MAK_2.1 has a major introgression on chromosome 2, and an additional one in the region of *fchl.9-fca.9-fcb.9* (36.8–64 cM/CMPSNP159-CMPSNP890, Additional file 3), which could be causing the effects on flesh color. Therefore, the occurrence of a flesh color QTL on chromosome 2 requires confirmation by the characterization of lines with single introgression.

The other ILs were all orange-fleshed, but some variation in the orange tone was observed. Fruits of MAK_6-1 and MAK_8-2 have a paler orange color, with higher Hl values (Figs. 12 and 5) (*fchl.6* and *fchl.8*) (Fig. 1, Additional file 5). The fruits of MAK_8-2 presented a yellow color in the internal rind, which was different from the green that is usually found in most ILs and in VED fruits (Fig. 12). This trait appeared in some fruits of other lines. It was scored as present or absent (CIR, color of the inner rind) and analyzed. Mainly MAK_8-2, but also MAK_6-1 and MAK_12-1, yielded fruits with yellow internal rind. The MAK_8-2 line has a single introgression (46.5–79.2 cM/CMPSNP281-CMPSNP1006), but both MAK_6-1 and MAK_12-1 have a second introgression, in addition to their main one, in common with MAK_8-2 in chromosome 8 (Additional file 3), suggesting that the CIR effect is due to the MAK introgression in chromosome 8 (*cir.8*).

Only one line presented a significant increase in the b* parameter in at least two environments (Fig. 5). MAK_2-3 have orange-fleshed fruits with increased b* values, with more yellowish flesh (Fig. 12), defining the *fcb.2* QTL (Fig. 1 and Additional file 5).

Carotenoid content has been studied in several previous works and has been reported to be independent of ripening behavior [48]. In these previous studies, QTLs for carotene content co-locate with the major flesh color genes *gf* (green flesh) and *wf* (white flesh), located in chromosomes 8 and 9, respectively. Classic studies indicate that these genes interact epistatically: *wf + −/gf + −* and *wf + −/gfgf* allelic combinations have orange flesh, *wfwf/gf + −* white flesh and *wfwf/gfgf* green flesh [51]. In our population, the effect of MAK introgressions containing these genes also altered fruit color. Fruits of MAK_9-2 (with a single introgression on chromosome 9 in the region of the *wf*) showed green flesh (Additional file 3 and Fig. 12). MAK fruits are white-fleshed, so this cultivar has the *gf* and *wf* allele combination that leads to white flesh (*wfwf/gf + gf+*). On the other hand, the VED cultivar has the orange allele for *wf* and the green allele for *gf* (*wf + wf+/gfgf*) [19]. Thus, MAK_9-2 would have the combination of *wfwf* (from MAK) and *gfgf* (from VED), leading to green-fleshed fruits. We also observed the epistatic interaction between the two genes in fruits of MAK_8-2. This line has a VED genotype in chromosome 9, *wf + wf+,* but contains a MAK introgression in chromosome 8 in the region of *gf*, *gf + gf+*. This combination is, as expected, orange-fleshed. However, the effect of the *gf* gene is not totally masked, as previously suggested, by the presence of *wf +* alleles, and the allelic combination found in MAK_8-2, *wf + wf+/gf + gf+*, resulted in a different orange phenotype than the VED allelic combination, *wf + wf+/gfgf*, with higher luminosity in the orange color, and a characteristic yellow color in the internal part of the rind*.* This combined effect of the *wf* and *gf* genes should be considered in Charentais breeding programs to recover the orange flesh/green inner rind phenotype characteristic of this type of melons (Fig. 12).

#### Sugars content

Sugar content is one of the most important traits for seed companies and producers, as it is related to the organoleptic value of the fruits. Melon germplasm can be divided into sweet and non-sweet melons, which has been attributed to variability in a sucrose accumulator gene [52], but a large number of QTLs have been detected in sugar accumulation traits, demonstrating that this trait has a very complex genetic control in melon [5].

Soluble solids content, SSC, measured in the three environments, presented variable, low-to-moderate, values of heritability (h^2^ = 0.08 to 0.5), with the genotype effect being relatively modest (16.8 % of the total variation) but higher than the environmental effect (3.3 %) (Additional file 4). An important G x E interaction was detected in SSC (15.9 %). The content of specific sugars (sucrose, fructose and glucose) was measured in one environment, and also had low heritability values (h^2^ = 0.29, 0.15, and 0.22, respectively for each sugar).

Despite the G × E interactions, fruits of the IL MAK_10-1 had significantly higher SSC values than VED in the three environments (12.5 to 24.4 %) (Fig. 6). This QTL, *ssc.10*, co-localizes with *suc.10,* which increases the sucrose amount of MAK_10.1 fruits by 27.7 % compared to VED. Sugar accumulation is independent of climacteric behavior; in fact, non-climacteric cultivars are among the sweetest melons. However, some climacteric melons, such as the Charentais VED, suffer a rapid decay of sugar content after reaching full maturity. The major QTL on chromosome 10 co-localized with the region in which the ripening process was delayed (*al.10-ff.10- ar.10*) (Fig. 1), so this higher sugar content could be related to the delay in the ripening process. Another line with significantly different sugar content than that of the recurrent parent was MAK_5-2, which yielded fruits with significantly higher sucrose (17.9 %) and lower glucose (−8.6 %) and fructose (−5.8 %) (*suc.5, gluc.5* and *fruct.5*) (Fig. 6 and Additional file 5). [53] defined a QTL in chromosome 5 that affects sugar content in the PI 161375 × “Piel de sapo” population, but in this case, the alleles of PI161375 reduced the sucrose content. To our knowledge, *suc.10* and *suc.5* are the first QTLs in which exotic alleles have been reported to increase the level of sucrose in melon.

Two other MAK regions were found to be involved in sugar variation. MAK_1-2 had significantly reduced sucrose content (−39.4 %) and higher levels of glucose (18.3 %) than VED (Fig. 6) (*suc.1*. and *gluc.1*) (Additional file 5). The introgression from MAK on chromosome 1 includes a Sulfur susceptibility gene [54]. Sulfur dust is used to control powdery mildew in greenhouses, causing severe leaf necrosis and consequently has major effects on fruit development. The fact that MAK carries the susceptible allele could lead to the reduction in fruit sugar content resulting from a pleiotropic effect of Sulfur susceptibility. A similar effect was found in MAK_11-2 with reduced sucrose (−15 %) and increased glucose (38 %) and fructose contents (22.3 %) (*suc.11*, *fruc.11* and *gluc.11*), but this line is not sulfur sensitive (Additional file 5, Fig. 6).

Variation of sugar accumulation in sweet melons is controlled by a large number of QTLs, which have been mapped in all chromosomes, but are more frequent in chromosomes 2, 3, 5 and 8. Our results confirm the large effect of the G × E interaction reported in previous studies [20], but despite this effect, we can confirm the region previously found in chromosome 5, and suggest the existence of additional regions in chromosomes 1, 10 and 11. The major effect found in chromosome 10, which could be related to a delay in ripening, is of interest for maintaining an optimal sugar level during the ripening process in Charentais melons. The fact that no clear co-localization of QTLs with genes involved in sugar metabolism has been reported up to now [8, 23] makes the identification of candidate genes difficult.

### Stability of QTL detection in backcrosses and ILs

The developed IL population was, as expected, more effective at detecting QTLs than the backcrosses. For example, none of the QTLs related to flowering and maturity time detected in the IL populations were detected in the backcross populations (Table 1). This result could be explained in part by the occurrence of a high G × E interaction (as observed for NMaF30, NFeF30 and Dmat, Additional file 4). A similar situation occurred with the QTLs related to fruit size, FW, FD and FL. However, in this case, the G × E interaction was not so important, suggesting that the results are more likely due to the different genetic structures of the populations. FW QTLs usually show an additive gene action [55], so the power to detect them in backcross populations is lower than in IL populations.

Conversely, the major QTL related to fruit shape, *fs.11,* was stable across populations (Table 1 and Additional file 5). This high stability of FS QTLs compared with FW may be explained by the common dominant gene action observed in melon FS QTLs [55], making their detection in backcrosses easier. A similar situation was found with QTL *ff.10,* which is involved in flesh firmness variation. This trait, just like fruit shape, had a very low environmental effect and an only moderate G × E interaction. In addition, the strong genotype effect found for the flesh color traits, associated with almost no G x E interaction, along with the dominance of the major genes reported to be involved in flesh color, might account for the stability of the *fchl.9, fca.9* and *fcb.9* QTLs.

We were also able to find several stable QTLs for SSC and specific sugars in chromosomes 1, 5 and 10 (Fig. 1, and Additional file 5). This stability may facilitate their introduction in breeding programs.

## Conclusion

This work presents the first collection of ILs in a cantaloupe genetic background. This strategy of obtaining pre-breeding lines with characteristics of interest will encourage breeding in Charentais melons, one of the most commercially important types. The 27 ILs, selected after several cycles of backcrossing, selfing and marker-assisted selection, represent most of the MAK genome, with an average of 1.3 introgressions per line. This IL collection, phenotyped in three different environments and genotyped with a medium-throughput platform, has allowed us to study important traits in this crop and their association to certain genomic regions. The QTL detection performed using this IL collection has been demonstrated to be more effective compared to other populations, such as backcrosses. In total, 47 QTLs, significant in at least two IL assays, have been identified for traits related to fruit quality. Many have been detected in this work for the first time, while others confirm previously reported QTLs. The results presented herein, related to flowering and maturity time traits, fruit morphology, ripening behavior, rind characteristics, flesh color and sugars content, will not only facilitate the knowledge of the genetic control of these traits, but have also provided interesting lines for breeding, such as the one with delayed climacteric ripening behavior and sweeter fruits, or the small-fruited lines. Further genotyping with new high-throughput methods, such as GBS (Genotyping by sequencing), and new subIL sets with smaller introgressions, will allow these results to be confirmed, and will expand our knowledge of the candidate genes underlying these interesting QTLs.

## Methods

### Plant material

The two parents used to generate the IL population were the French cultivar Vedrantais (VED) (*C. melo* subsp. *melo* var. *cantalupensis*, Charentais type) as recurrent parent and the Japanese accession Ginsen makuwa (MAK) (*C.melo* subsp. *agrestis* var. makuwa) as donor parent. VED and MAK were selected from a core germplasm collection established on the framework of a previous project MELRIP 2007–2010 [7]. Accessions of these core collection were multiplied and conserved in the genebank at COMAV-UPV. The MAK parent derived from accession PI 420176 (kindly provided by USDA, NPGS) after several selfing cycles. Both VED and MAK were morphologically characterized previously, along with a larger collection of melons, to confirm their classification in the corresponding horticultural group [8].

VED represents one of the most important market classes of cantaloupe melons. It produces medium-size, oval-to-round, sutured and orange-fleshed fruits, with a typical climacteric ripening behavior (with an autocatalytic production of ethylene during ripening), that are aromatic and have a medium sugar content. MAK fruits are small and oval. The flesh is white, sweet with little aroma, and shows a certain level of climacteric ripening behavior (Additional file 1). Makuwa cultivars are the only melon landraces belonging to subspecies *agrestis* that have a flesh sugar content similar to or even higher than that of sweet melons from ssp.*melo* [8, 37].

### Breeding scheme

The F1 generation derived from the cross between VED and MAK was backcrossed with the recurrent VED parent to generate the BC1 population. Fifteen BC1 plants were then backcrossed with the recurrent parent thus producing fifteen BC2 families. A total of 420 BC2 plants (28 per BC2 family) were genotyped at the seedling stage using a Sequenom iPLEX® Gold MassARRAY with a set of 154 SNPs evenly distributed throughout the melon genome (see details below). A subset of the 420 genotyped BC2 seedlings was selected according to their genotype: those having the highest proportion of the recurrent (VED) genome, and which contained MAK introgressions that, together, covered the entire donor (MAK) genome at least twice. The selected BC2 plants were grown in the greenhouse and backcrossed to construct the BC3 population.

A total of 363 BC3 plants were genotyped with the same set of SNPs, at the seedling stage, and were selected according to their genotype to produce the next generations. One set of selected BC3 plants, which had three or fewer MAK introgressions, was grown at the greenhouse and selfed. The BC3S1 offsprings were genotyped with the SNPs in the corresponding target introgressions by High Resolution Melting (HRM) [56] in order to finally select plants with single homozygous introgressions. The HRM genotype of the selected BC3S1 plants was validated with the Sequenom array. The number of BC3S1 plants that were necessary to screen in each progeny to obtain single introgression lines (*p* = 0.95) was calculated from the binomial distribution of the allele segregation as previously described [28]. Another set of selected BC3 plants, with four or more MAK introgressions, was also used to generate plants with single introgressions, but in two steps, as the number of progenies that needed to be screened by direct selfing to separate the introgressions was too high in this case. Then, these BC3 were first backcrossed and the resulting BC4 plants with single or double introgressions, identified by the HRM analysis, were then selfed to generate the BC4S1.

In some cases, the BC3S1 and BC4S1 that were ultimately selected were selfed again to remove a few remaining heterozygous markers and to produce seeds for the characterization assays. A first set of 27 ILs (including BC3S1, BC4S1, BC3S2 and BC4S2 plants) with single or double homozygous introgressions was characterized in the present paper. The genotype of these lines was validated again using the Sequenom array. This is a medium-sized IL population, 2 to 3 lines per chromosome, but it covers most of the MAK genome, having mostly single introgressions, and represents a set good enough for evaluating the breeding potential of the current population.

### Markers and genotyping methods

Genomic DNA was extracted from young leaves following the [57] method. The extracted DNA was dissolved in Milli-Q water, and the final concentration was adjusted to 10 ng/μl for the Sequenom and 30 ng/μl for the HRM genotyping.

SNPs were selected from those previously mapped in the melon genetic map used to anchor the first version of the melon genome [9]. This map was constructed using a mapping population derived from the cross *C.melo* ssp *melo* var *inodorus* Piel de sapo and *C.melo* ssp *agrestis* var *conomon* Songwhan Charmi. The available map included 580 SNPs located in 249 different genetic positions (20.8 per chromosome, with an average genetic distance between markers of 4.1 cM, from 0.8 to 20.1 cM). A set of 144 of these mapped SNPs was selected according to their genetic positions, and 135 met the multiplexing requirements of Agena’s Assay Designer used for the Sequenom iPLEX ® Gold MassARRAY and could be implemented in the genotyping assay (representing 128 genetic positions, with an average distance of 8.2 cM, from 1.6 to 22.4 cM). Three of these markers could not be called accurately in the Sequenom assay, and thirteen failed to show the expected polymorphism between the IL parents (VED and MAK) (Additional file 2). An additional set of 35 SNPs was also added to the Sequenom array. These were selected from an SNP collection that had been validated by Sequenom in the study by [8] and had been generated in a previous resequencing study, using VED and MAK among other melon genotypes [3] (Additional file 2). Most were located in candidate genes involved in sugar and ethylene metabolism, and some were found to be associated with these traits in [8]. All worked with the Sequenom assay and were polymorphic between VED and MAK. The final Sequenom array, with a total of 154 working and polymorphic SNPs, was used to genotype the full BC2 and BC3 populations, as well as the selected BC3S1 and the set of ILs selected for phenotyping. The Sequenom genotyping was done at the Epigenetics and Genotyping laboratory located at the Central Research Unit of the Faculty of Medicine (UCIM) belonging to the University of Valencia (Spain).

HRM genotyping with different subsets of these markers was also used to accelerate the selection and fixation of target introgressions during the construction of the IL population in several specific BC4, BC3S1, BC4S1, BC3S2 and BC4S2 offsprings. The PRIMER3 software program [58] was used to design the oligonucleotides for the HRM analysis. A total of 97 SNPs, out of the 154 employed in the full Sequenom platform, were adapted for HRM analysis.

### Agronomic evaluation and characters measured

The intermediate backcross populations generated during the development of the IL population, BC2, BC3 and BC3S1, were fully genotyped at the seedling stage as described previously. Some of these genotyped plants were selected on the basis of their genotype (those with the highest proportion of VED genome and which contained MAK introgressions that, together, represented the entire MAK genome) and were transplanted to the greenhouse for phenotyping and for the generation of the next generation. A total of seventy-five BC2, one hundred BC3 and ninety–six BC3S1 plants were phenotyped in 2011, 2012 and 2013, respectively, during the spring-summer growing cycle at the greenhouse facilities of the Polytechnic University of Valencia (Valencia, Spain).

Additionally, twenty-seven lines of the final IL collection were evaluated in three trials, all conducted under greenhouse conditions. Two were conducted in spring-summer of 2014 and 2015 in the facilities of the Fundacion Cajamar in Paiporta (Valencia, Spain) (Paip14 and Paip15), and the third at the Polytechnic University of Valencia during the spring-summer of 2015 (UPV15). Each assay included six to eight plants of each of the 27 ILs that were grown in a fully randomized design along with five to ten plants of each parental line (VED and MAK). Flowers were hand-pollinated in Paip14 and UPV15, and insect pollination was used in Paip15 to produce two fruits per plant.

The UPV’s greenhouse conditions used for all the backcross and for the UPV15 IL phenotyping assays were as follows: growing cycle from March to July in a glass greenhouse with automatic control of temperature with cooler and automatic window aperture (with a temperature range of 8 to 15 °C and of 25 to 32 °C, minimum and maximum during the whole growing cycle). Plants were grown in 15-L pots with a substrate of 100 % coconut fiber.

The greenhouse conditions of the Fundacion Cajamar, used for the Paip14 and Paip15 IL phenotyping assays were as follows: growing cycle from March to July in a glass greenhouse with automatic control of temperature with cooler and automatic window aperture (with a temperature range of 10 to 25/10 to 20 and of 25 to 37/18 to 35, minimum and maximum during the whole growing cycle for Paip2014/2015, respectively). Plants were grown in substrate bags of 29 kg (70 % coconut fiber and 30 % coconut chips). In both cases, nutrients were provided through the irrigation system and pruning was done manually when necessary to regulate vegetative growth and flowering.

Each plant was phenotyped for traits related to flowering, days to maturity and fruit quality. Regarding flowering, the number of male and female flowers 30 days after the opening of the first female flower on each plant was counted (NMaF30and NFeF30). Also, the days to maturity (DMat), which is the number of days from the date of hand-pollination to the harvest, were counted for each fruit. Two fruits per plant were set and characterized at full maturity. The following traits were measured for each fruit: fruit weight (FW in grams, with digital scale), fruit length and diameter (FL, FD in mm, with graduated rule), fruit shape index (FS, as the ratio of fruit length to fruit diameter), cavity width (CW, as the ratio of the width of the seminal cavity to the fruit diameter), flesh and rind thickness (Fth, Rth in mm, with electronic digital caliper, I.C.T, S.L., La Rioja, España), rind and flesh firmness (RF, FF, measured as kg/cm^2^ with a fruit pressure tester, FT 327, with a plunger diameter of 8 mm, Alfonsine, Italy), the formation of an abscission layer, the external aroma of the whole fruit and the netting occurrence (AL, AR, NET, scored visually as 0, absent and 1, present), flesh color measured with a CR-400 colorimeter, Konica Minolta, Inc., Tokyo, Japan (coordinates Hunter Lab. L* express luminosity (L = 0 black and L = 1 white), a* expresses the color direction between red (positive) and green (negative) and b* expresses the color direction between yellow (positive) and blue (negative)) (FCHl, FCa, FCb), color of the inner rind, CIR (scored visually as 0, green and 1, yellow) and soluble solids concentration (SSC) (measured as °Brix from drops of juice with a hand-held “Pocket” refractometer (PAL-α), Atago CO., LTD, Tokyo, Japan). Flesh firmness, color and total soluble solids were measured at two points in the equatorial region of the mesocarp. In addition to to SSC, sucrose, glucose and fructose (SUC, GLUC and FRUC) were quantified (μmol/gFW eq. Hexose) in fruits of the BC3, BC3S1 and Paip14 assays, where flesh samples were taken from the same regions in the equatorial slice of the fruit used for firmness, flesh color, Brix and pH measurements. Flesh tissue was shock frozen in liquid nitrogen and ground to homogeneity. Aliquots of about 20 mg were weighted and sent to INRA Bordeaux on dry ice for analysis. Metabolites were extracted by ethanolic fractionation as in [59]. Glucose, fructose and sucrose were determined enzymatically in the ethanolic supernatant as in [60]. Assays were performed in 96-well polystyrene microplates using Starlet pipetting robots (Hamilton, Villebon-sur-Yvette, France), and absorbance was read at 340 nm in MP96 microplate readers (SAFAS, Monaco).

### Association analysis in backcross families

The phenotypic and genotypic data of the backcross populations (BC2, BC3 and BC3S1) were used to detect significant associations between markers and phenotypic values. The association analysis was performed using TASSEL v. 5 (Trait Analysis by aSSociation, Evolution and Linkage) [61] for both approaches: a general linear model (GLM) and a mixed linear model (MLM) analysis using a kinship matrix as cofactor to avoid spurious associations due to relatedness and population structure. Associations were considered statistically significant at *p* < 0.005. We considered as associations those that were significant with both the GLM and MLM approaches, or those that were significant with the GLM and which were later validated in the IL analysis. Genotype effects and percent of phenotypic variance explained by each marker were also calculated.

### QTL analysis in introgression lines

IL data in the three environments were analyzed using an analysis of variance (ANOVA) that was performed in order to examine the effects of genotype, environment and genotype-×-environment interaction. Estimation of heritability (h^2^ = VarG/(VarG + VarE) was performed for each trait and environment by calculating the variance components from the mean squares (MS) within and between the ILs with a hierarchical ANOVA (MSbetween = VarE + n VarG and MSwithin = VarE, where VarG = genotypic variance, VarE = environmental variance, and n = number of plants per IL).

Furthermore, the mean of every IL in each environment was compared to the control VED mean with the Dunnett’s test at *p* < 0.05. QTLs for each trait in the MAK introgression were considered to exist in those lines that had means that were significantly different from VED in at least two localities.

## Abbreviations

AL, abscission layer; AR, external aroma; BC, backcross; CIR, color of the inner rind; CMV, Cucumber Mosaic Virus; CW, cavity width; DMat, days to maturity; E, environment; FCa, flesh color Hunter Lab. a; FCb, flesh color Hunter Lab. b; FCHl, flesh color Hunter Lab. L; FD, fruit diameter; FF, flesh firmness; FL, fruit length; FRUC, fructose; FS, fruit shape; FW, fruit weight; G, genotype; gf, green flesh; GLUC, glucose; IL, introgression lines; LG, linkage group; MAK, Makuwa; MAS, marker-assisted selection; Net, netting; NFeF30, number of female flowers 30 days after the opening of the first female flowering on each plant; NGS, next generation sequencing; NMaF30, number of male flowers 30 days after the opening of the first female flowering on each plant; QTL, quantitative trait locus; RTh, rind thickness; SNP, Single Nucleotide Polymorphism; SSC, soluble solid concentration; SSR, Single Sequence Repeats; SUC, sucrose; TILLING, targeted induced local lesions in chromosome; VED, Vedrantais; wf, white flesh

## Additional files

Additional file 1: Fruits of the two parents used to generate the IL population and the corresponding F1. From left to right: the cultivar Vedrantais (VED) (*C. melo* subsp. *melo* var. *cantalupensis*, Charentais type) used as recurrent parent, Ginsen makuwa (MAK) (*C. melo* subsp. *agrestis* var. *makuwa*) used as donor parent, and their F1. (PPTX 876 kb) Additional file 2: SNPs used for the construction of the IL population. Background SNPs Sequenom-HRM: These were selected from those previously mapped in the melon genetic map used to anchor the first version of the melon genome [9]. The map position is indicated according to the map used to anchor the genome [9] as well as a new version of the genetic map [11]. The physical position in the last version of the melon genome (v3.5.1) available at [10] is indicated. The SNP and flanking sequence is included for all the markers used in the Sequenom assay, and the primers for those that were adapted to the HRM genotyping procedure are also provided. Markers that could not be accurately called in the Sequenom assay and that failed to show the expected polymorphism between the IL parents (VED and MAK) are marked as failed markers (f). SNPs in candidates: Additional set of SNPs located in candidate genes reported to be involved in sugar and ethylene metabolism. These markers were validated in [8] and had been generated in a previous resequencing study, using VED and MAK among other melon genotypes [3]. The physical position in the last version of the melon genome (v3.5.1) available at [10] is indicated. The SNP and flanking sequence is included for all the markers used in the Sequenom assay. (XLSX 39 kb) Additional file 3: Graphical genotype of the 27 ILs selected in this study for agronomic characterization in three trials. Rows from top to bottom: marker names, melon chromosome, genetic position according to [9] for the background markers (the SNPs located in candidate genes are not mapped and are located according to their physical position indicated in Additional file 2), and the SNP allele (VED/MAK). Green boxes indicate MAK homozygous introgressions, and blue lines VED genetic background. (XLSX 30 kb) Additional file 4: Mean, standard deviation (SD) and range values of the number of male and female flowers 30 days after the opening of the first flower (NMaF30 and NFe30), days to maturity (DMat), fruit weight (FW), fruit length (FL), fruit diameter (FD), fruit shape (FS), cavity width (CW), flesh firmness (FF), presence of abscission layer (AL), aroma (AR), rind thickness (RTh), netting (Net), flesh color parameters (FCHL, FCa and FCb), color of the inner rind (CIR), soluble solids content (SSC), and sucrose, glucose and fructose content (SUC, GLUC and FRUC) of both parents, VED and MAK, their F1 and the IL population assayed in three experiments. In the VED and MAK data, asterisks in rows indicate significant mean differences between trials (*p* < 0.05), and in the columns between parents; ns (not significant differences), na (not available). Estimation of heritability (h^2^ = VarG/(VarG + VarE) was performed for each trait and environment by calculating the variance components from the mean squares (MS) within and between ILs with an ANOVA (MSbetween = VarE + n VarG and MSwithin = VarE, where VarG = genotypic variance, VarE = environmental variance, and n = number of plants per IL). Data of the ILs in the three environments were analyzed using a two-factor ANOVA that was performed to examine the effect of genotype, environment and genotype-x-environment interaction. The percentage of variance explained by each effect (genotype, environment and the interaction) is indicated (**p* < 0.05, ***p* < 0.001 and ns (no significant differences)). (DOCX 43 kb) Additional file 5: List of all QTLs detected with the Dunnet’s test in at least two of the three trials performed with the selected IL collection. Abbreviated trait name as in Additional file 4, QTL name, chromosome, QTL position (cM) and flanking markers, according to the phenotype of overlapping lines, number of trials in which the QTL was detected, MAK effect relative to the VED parental (%) with positive/negative effects indicating that MAK alleles increase/decrease the value of the trait, IL introgression position (cM) and flanking markers. (XLSX 15 kb) Additional file 6: Correlation coefficient (*p* < 0.05) of the traits used for phenotyping the ILs in the three trials: Paip15, UPV15 and Paip14. Dark grey represents positive correlations and light gray represents negative correlations. Abbreviated trait name as in Additional file 4. (XLSX 15 kb)
