# Supplementary material for: A new genomic library of melon introgression lines in a cantaloupe genetic background for dissecting desirable agronomical traits
Source: BMC Plant Biol. 2016 Jul 8;16:154. doi: 10.1186/s12870-016-0842-0 (PMC4938994; doi:10.1186/s12870-016-0842-0)
Supplement: Additional file 1: — Fruits of the two parents used to generate the IL population and the corresponding F1. From left to right: the cultivar Vedrantais (VED) (C. melo subsp. melo var. cantalupensis, Charentais type) used as recurrent parent, Ginsen makuwa (MAK) (C. melo subsp. agrestis var. makuwa) used as donor parent, and their F1. (PPTX 876 kb) [file 12870_2016_842_MOESM1_ESM.pptx]

## Slide 1
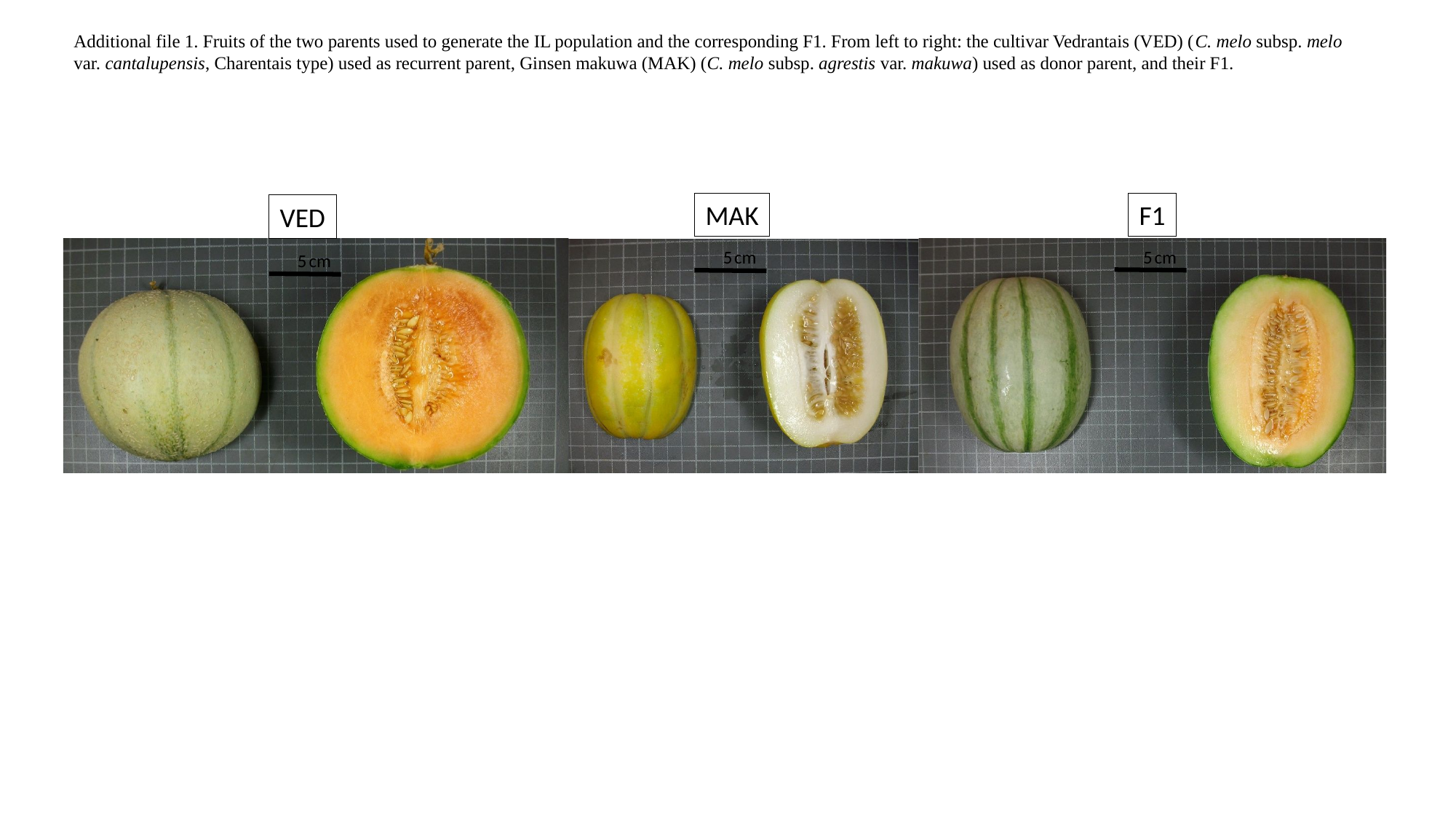

Additional file 1. Fruits of the two parents used to generate the IL population and the corresponding F1. From left to right: the cultivar Vedrantais (VED) (C. melo subsp. melo var. cantalupensis, Charentais type) used as recurrent parent, Ginsen makuwa (MAK) (C. melo subsp. agrestis var. makuwa) used as donor parent, and their F1.
MAK
F1
VED
5 cm
5 cm
5 cm
