# Supplementary material for: A new genomic library of melon introgression lines in a cantaloupe genetic background for dissecting desirable agronomical traits
Source: BMC Plant Biol. 2016 Jul 8;16:154. doi: 10.1186/s12870-016-0842-0 (PMC4938994; doi:10.1186/s12870-016-0842-0)
Supplement: Additional file 4: — Mean, standard deviation (SD) and range values of the number of male and female flowers 30 days after the opening of the first flower (NMaF30 and NFe30), days to maturity (DMat), fruit weight (FW), fruit length (FL), fruit diameter (FD), fruit shape (FS), cavity width (CW), flesh firmness (FF), presence of abscission layer (AL), aroma (AR), rind thickness (RTh), netting (Net), flesh color parameters (FCHL, FCa and FCb), color of the inner rind (CIR), soluble solids content (SSC), and sucrose, glucose and fructose content (SUC, GLUC and FRUC) of both parents, VED and MAK, their F1 and the IL population assayed in three experiments. In the VED and MAK data, asterisks in rows indicate significant mean differences between trials (p < 0.05), and in the columns between parents; ns (not significant differences), na (not available). Estimation of heritability (h2 = VarG/(VarG + VarE) was performed for each trait and environment by calculating the variance components from the mean squares (MS) within and between ILs with an ANOVA (MSbetween = VarE + n VarG and MSwithin = VarE, where VarG = genotypic variance, VarE = environmental variance, and n = number of plants per IL). Data of the ILs in the three environments were analyzed using a two-factor ANOVA that was performed to examine the effect of genotype, environment and genotype-x-environment interaction. The percentage of variance explained by each effect (genotype, environment and the interaction) is indicated (*p < 0.05, **p < 0.001 and ns (no significant differences)). (DOCX 43 kb) [file 12870_2016_842_MOESM4_ESM.docx]

Aditional file 4. Mean, standard deviation (SD) and range values of number of male and female flowers 30 days after the opening of the first flower (NMaF30 and NFe30), days to maturity (DMat), fruit weight (FW), fruit length (FL), fruit diameter (FD), fruit shape (FS), cavity width (CW), flesh firmness (FF), presence of abscission layer (AL), aroma (AR), rind thickness (RTh), netting (Net), flesh color parameters (FCHL, FCa and FCb), color of the inner rind (CIR), soluble solids content (SSC), and sucrose, glucose and fructose content (SUC, GLUC and FRUC) of both parents, VED and MAK, their F1 and the ILs population assayed in three experiments. In VED and MAK data asterisks in rows indicate significant mean differences between trials (p<0.05), and in columns between parents; ns (non-significant differences), na (not available). Estimation of heritability (h^2^=VarG/(VarG+VarE) was performed for each trait and environment by calculating the variance components from the mean squares (MS) within and between ILs with an ANOVA (MSbetween= VarE + n VarG and MSwithin=VarE, where VarG = genotypic variance, VarE = environmental variance, and n=number of plants per IL). Data of the ILs in the three environments were analyzed using a two-factor ANOVA that was performed to examine the effect of genotype, environment, and genotype-x-environment interaction. The percentage of variance explained by each effect (genotype, environment and the interaction) is indicated (*p<0.05, **p<0.001 and ns (non-significant differences).

| Trait | Trials | VED | | MAK | |  | F1 | | ILs | | | | Heritability | % variance | | |
| --- | --- | --- | --- | --- | --- | --- | --- | --- | --- | --- | --- | --- | --- | --- | --- | --- |
|  |  | Mean | SD | Mean | SD |  | Mean | SD | Mean | SD | | Range |  | Genotype | Environment | Interaction (G x E) |
| NMaF30 | Paip14 | 9.2 | 5.1 | 6 | 1.4 | ns | na | na | 7.6 | 3.2 | | 2 – 14 | 0.26 |  |  |  |
|  | Paip15 | 2.5 | 0.7 | na | na | na | 5 | 2.1 | 6.0 | 2.5 | | 1.1 – 12.1 | 0.36 | 11.2** | 23.9** | 19.6** |
|  | UPV15 | 11 | 1.4 | 11 | 1.1 | ns | 6 | 1.2 | 17.8 | 9.2 | | 1.6 – 36.1 | 0.4 |  |  |  |
|  |  | ns |  | * |  |  | na |  | * |  | |  |  |  |  |  |
| NFeF30 | Paip14 | 2.5 | 1.2 | 3.2 | 1.1 | * | na | na | 2.6 | 1.3 | | 0 – 5.5 | 0.15 |  |  |  |
|  | Paip15 | 1.2 | 1.1 | na | na | na | 2 | 1.0 | 1.6 | 1.1 | | 0 – 3.9 | 0.26 | 9.8** | 3.0** | 18.3** |
|  | UPV15 | 1.3 | 1.0 | 4.1 | 1.8 | * | 0 | 0 | 2.5 | 1.2 | | 0.62 – 5 | 0.17 |  |  |  |
|  |  | * |  | ns |  |  | na |  | * |  | |  |  |  |  |  |
| DMat | Paip14 | 43.3 | 3.0 | na | na | na | na | na | 42.3 | 5.7 | | 32 - 52.1 | 0.48 |  |  |  |
|  | Paip15 | na | na | na | na | na | na | na | na | na | | na | na | 25.7** | 24** | 10.1** |
|  | UPV15 | 40.0 | 2.0 | na | na | na | na | na | 36.1 | 3.5 | | 27 - 43.6 | 0.26 |  |  |  |
|  |  | ns |  |  |  |  |  |  | * |  | |  |  |  |  |  |
| FW | Paip14 | 614.1 | 140.3 | 187.1 | 42.8 | * | na | na. | 680.6 | 154.9 | | 985.5 - 366.9 | 0.52 |  |  |  |
|  | Paip15 | 719.8 | 39.6 | 184.0 | 15.6 | * | 363.5 | 140.7 | 685.8 | 148.9 | | 994.4 - 383.12 | 0.43 | 26.6** | 18.8** | ns |
|  | UPV15 | 933.9 | 210.0 | 360.7 | 66.1 | * | 595.0 | 7.0 | 941.8 | 195.3 | | 1358.3 - 579.1 | 0.38 |  |  |  |
|  |  | * |  | * |  |  | ns |  | * |  | |  |  |  |  |  |
| FL | Paip14 | 98.6 | 5.05 | 77.8 | 11.1 | * | na | na | 103.5 | 10.1 | | 123.3 - 75. | 0.55 |  |  |  |
|  | Paip15 | 104.1 | 4.4 | 71.2 | 6.9 | * | 103.0 | 7.0 | 103.4 | 11.2 | | 123.1 - 8 | 0.49 | 30.5** | 20.2** | 6.2* |
|  | UPV15 | 112.7 | 9.3 | 96.8 | 8.1 | * | 117.5 | 3.5 | 117.8 | 11.2 | | 143.1 - 99.6 | 0.41 |  |  |  |
|  |  | * |  | * |  |  | ns |  | * |  | |  |  |  |  |  |
| FD | Paip14 | 107.6 | 8.9 | 64 | 3.2 | * | na | na | 109.9 | 8.2 | | 122.5 - 91.0 | 0.44 |  |  |  |
|  | Paip15 | 111.1 | 2.1 | 68 | 1.2 | * | 88.5 | 3.5 | 110.1 | 8.5 | | 122.9 - 89.8 | 0.42 | 22.6** | 19.0** | 8.1* |
|  | UPV15 | 126.3 | 9.2 | 81 | 7.4 | * | 98.5 | 0.70 | 123.6 | 8.2 | | 141 - 106.12 | 0.43 |  |  |  |
|  |  | * |  | * |  |  | ns | na | * |  | |  |  |  |  |  |
| FS | Paip14 | 0.92 | 0.04 | 1.2 | 0.17 | * | na | na | 0.94 | 0.05 | | 1.0 - 0.83 | 0.48 |  |  |  |
|  | Paip15 | 0.94 | 0.02 | 1.0 | 0.11 | ns | 1.1 | 0.03 | 0.94 | 0.06 | | 1.0 - 0.85 | 0.43 | 37.2** | 2.8** | 7.7* |
|  | UPV15 | 0.89 | 0.05 | 1.2 | 0.14 | * | 1.1 | 0.02 | 0.95 | 0.06 | | 1.0 - 0.83 | 0.54 |  |  |  |
|  |  | ns | 0,91 | ns |  |  | ns |  | ns |  | |  |  |  |  |  |
| CW | Paip14 | 0.49 | 0.04 | 0.66 | 0.02 | * | na | na | 0.49 | 0.02 | | 0.53 - 0.44 | 0.08 |  |  |  |
|  | Paip15 | 0.42 | 0.01 | 0.58 | 0.03 | * | 0.58 | 0.01 | 0.46 | 0.03 | | 0.54 - 0.41 | 0.29 | 16.3** | 2.4** | 11.7* |
|  | UPV15 | 0.45 | 0.04 | 0.59 | 0.04 | * | 0.46 | 0.07 | 0.47 | 0.03 | | 0.54 - 0.39 | 0.23 |  |  |  |
|  |  | * |  | * |  |  | * |  | * | |  |  |  |  |  |  |
| FF | Paip14 | 2.3 | 0.65 | 2.05 | 0.93 | ns | na | na | 3.1 | | 0.92 | 6.2 - 1.4 | 0.37 |  |  |  |
|  | Paip15 | 1.9 | 0.35 | 2.3 | 0.95 | ns | 4.2 | 0.57 | 2.7 | | 0.76 | 4.7 - 1.5 | 0.30 | 29.2** | 2.2** | 9.1* |
|  | UPV15 | 2.0 | 0.73 | 2.8 | 1.3 | * | 4.2 | 0.57 | 2.5 | | 0.85 | 4.5 - 1.2 | 0.50 |  |  |  |
|  |  | ns |  | * |  |  | ns |  | * | |  |  |  |  |  |  |
| AL | Paip14 | 1.00 | 0.00 | 0.00 | 0.00 | * | na | na | 0.9 | | 0.21 | 1 – 0 | 0.33 |  |  |  |
|  | Paip15 | 1.00 | 0.00 | 0.00 | 0.00 | * | 1.00 | 0.00 | 0.93 | | 0.16 | 1 – 0 | 0.44 | 35.9** | 1.6** | 11.5* |
|  | UPV15 | 1.00 | 0.00 | 0.00 | 0.00 | * | 1.00 | 0.00 | 0.83 | | 0.26 | 1 – 0 | 0.5 |  |  |  |
|  |  | ns |  | ns |  |  | ns |  | ns | |  |  |  |  |  |  |
| AR | Paip14 | 1.00 | 0.00 | 1.00 | 0.00 | ns | na | na | 0.74 | | 0.28 | 1 – 0 | 0.34 |  |  |  |
|  | Paip15 | 1.00 | 0.00 | 1.00 | 0.00 | ns | 1.00 | 0.00 | 0.8 | | 0.25 | 1 – 0 | 0.4 | 31.6** | ns | 11.2* |
|  | UPV15 | 1.00 | 0.00 | 1.00 | 0.00 | ns | 1.00 | 0.00 | 0.7 | | 0.28 | 1 – 0 | 0.41 |  |  |  |
|  |  | ns |  | ns |  |  | ns |  | ns | |  |  |  |  |  |  |
| RTh | Paip14 | 4.8 | 1.0 | 2.0 | 0.99 | * | na | na | 4.9 | | 0.87 | 6.4 - 3.1 | 0.33 |  |  |  |
|  | Paip15 | 3.2 | 0.16 | 1.0 | 0.28 | * | 4.5 | 0.13 | 4.2 | | 0.92 | 6.1 - 1.3 | 0.49 | 30** | 8.9** | 13** |
|  | UPV15 | 4.7 | 1.06 | 2.1 | 1.3 | * | 6.3 | 1.2 | 5.2 | | 1.4 | 8.6 - 2.8 | 0.4 |  |  |  |
|  |  | * |  | ns |  |  | ns |  | * | |  |  |  |  |  |  |
| Net | Paip14 | 1.00 | 0.00 | 0.00 | 0.00 | * | na | na | 0.82 | | 0.22 | 1 – 0 | 0.22 |  |  |  |
|  | Paip15 | 1.00 | 0.00 | 0.00 | 0.00 | * | 0.00 | 0.00 | 0.85 | | 0.26 | 1 – 0 | 0.53 | 35.8** | 2.8** | ns |
|  | UPV15 | 1.00 | 0.00 | 0.00 | 0.00 | * | 0.00 | 0.00 | 0.7 | | 0.3 | 1 – 0 | 0.44 |  |  |  |
|  |  | ns |  | ns |  |  | ns |  | ns | |  |  |  |  |  |  |
| FCHl | Paip14 | 55.3 | 4.8 | 59.0 | 9.3 | ns | na | na | 55.6 | | 6.2 | 72.9 - 43.4 | 0.32 |  |  |  |
|  | Paip15 | 52.2 | 6.0 | 67.2 | 1.1 | * | 63.8 | 1.5 | 55.4 | | 4.7 | 64.6 - 42.8 | 0.36 | 29.1** | 7.9** | 9.5* |
|  | UPV15 | 53.4 | 8.1 | 67.9 | 4.9 | * | 64.8 | 0.12 | 59.8 | | 3.3 | 68.6 - 54.3 | 0.3 |  |  |  |
|  |  | * |  | * |  |  | ns |  | * | |  |  |  |  |  |  |
| FCa | Paip14 | 10.9 | 3.8 | -2.6 | 1.4 | * | na | na | 9.7 | | 5.4 | 14.7 - (-7.0) | 0.75 |  |  |  |
|  | Paip15 | 10.0 | 1.5 | -1.8 | 0.19 | * | 1.4 | 2.1 | 11.6 | | 6.4 | 16.7 - (-11.4) | 0.6 | 75.7** | 1.5** | 3.6** |
|  | UPV15 | 12.5 | 3.0 | -2.3 | 0.82 | * | 5.1 | 2.7 | 10.4 | | 5.0 | 14.0 - (-6.7) | 0.83 |  |  |  |
|  |  | * |  | ns |  |  | ns |  | * | |  |  |  |  |  |  |
| FCb | Paip14 | 26.5 | 3.7 | 11.4 | 1.4 | * | na | na | 23.6 | | 3.2 | 34.5 - 15.9 | 0.43 |  |  |  |
|  | Paip15 | 21.9 | 0.19 | 9.9 | 0.43 | * | 21.5 | 1.0 | 23.7 | | 2.0 | 27.5 - 19.1 | 0.56 | 32.3** | ns | ns |
|  | UPV15 | 22.9 | 2.5 | 10.5 | 1.5 | * | 22.1 | 1.2 | 23.7 | | 1.7 | 26.6 - 18.7 | 0.57 |  |  |  |
|  |  | * |  | ns |  |  | ns |  | ns | |  |  |  |  |  |  |
| CIR | Paip14 | 0 | 0 | 1 | 0 | * | 0 | 0 | 0.06 | | 0.2 | 1 – 0 | n.a. |  |  |  |
|  | Paip15 | 0 | 0 | 1 | 0 | * | 0 | 0 | 0.10 | | 0.30 | 1 – 0 | 0.86 | 55.5** | 0.7** | 9.5** |
|  | UPV15 | 0 | 0 | 1 | 0 | * | 0 | 0 | 0.13 | | 0.26 | 1 – 0 | 0.65 |  |  |  |
|  |  | ns |  | ns |  |  | ns |  | * | |  |  |  |  |  |  |
| SSC | Paip14 | 11.4 | 1.1 | 11.8 | 2.6 | ns | na | na | 11.9 | | 1.1 | 14.2 - 10.2 | 0.08 |  |  |  |
|  | Paip15 | 11.9 | 0.55 | 11.3 | 0.55 | ns | 14.1 | 0.84 | 11.9 | | 1.4 | 13.7 - 7.1 | 0.5 | 16.8** | 3.3** | 15.9** |
|  | UPV15 | 10.4 | 1.8 | 12.0 | 1.7 | * | 9.8 | 0.42 | 9.0 | | 1.5 | 13.4 - 6.4 | 0.17 |  |  |  |
|  |  | ns |  | ns |  |  | * |  | * | |  |  |  |  |  |  |
| SUC  GLUC  FRUC | Paip14 | 248.2 | 139.8 | 232.7 | 47.6 | ns | na | na | 236.3 | | 43.7 | 334.5 – 139.5 | 0.29 |  |  |  |
|  | Paip14 | 85.9 | 17.5 | 46.0 | 9.9 | * | na | na | 101.1 | | 17.9 | 135.6 – 60.2 | 0.15 |  |  |  |
|  | Paip14 | 87.5 | 21.0 | 50.9 | 3.2 | ns | na | na | 99.1 | | 13.2 | 129.2 – 73.2 | 0.22 |  |  |  |
